# Trans-Oral Video-Assisted Neck Surgery (TOVANS). A new transoral technique of endoscopic thyroidectomy with gasless premandible approach

**DOI:** 10.1007/s00464-012-2588-6

**Published:** 2012-11-21

**Authors:** Akihiro Nakajo, Hideo Arima, Munetsugu Hirata, Tadao Mizoguchi, Yuko Kijima, Shinichiro Mori, Sumiya Ishigami, Shinichi Ueno, Heiji Yoshinaka, Shoji Natsugoe

**Affiliations:** Department of Surgical Oncology, Digestive Surgery, Breast and Thyroid Surgery, Kagoshima University, 8-35-1 Sakuragaoka, Kagoshima, 890-8520 Japan

**Keywords:** Transoral, Video assisted neck surgery, Endoscopic thyroidectomy, Minimally invasive thyroidectomy, Endoscopic lymphadenectomy, TOVANS

## Abstract

**Background:**

Endoscopic thyroidectomy is a well-established surgical technique. We have been utilizing precordial video-assisted neck surgery (VANS) with a gasless anterior neck skin lifting method. Recently, natural orifice transluminal endoscopic surgery (NOTES) has generated excitement among surgeons as potentially scar-free surgery. We developed an innovative gasless transoral technique for endoscopic thyroidectomy that incorporated the concept of NOTES in a VANS-technique.

**Methods:**

Incision was made at the vestibulum under the inferior lip. From the vestibulum to the anterior cervical region, a subplatysmal tunnel in front of the mandible was created and cervical skin was lifted by Kirschner wires and a mechanical retracting system. This method without CO_2_ insufflation created an effective working space and provided an excellent cranio-caudal view so that we could perform thyroidectomy and central node dissection safely.

**Results:**

Beginning with our first clinical application of TOVANS in September 2009, we have performed eight such procedures. Three of the eight patients had papillary microcarcinoma and received central node dissection after thyroidectomy. All patients began oral intake 1 day after surgery. The sensory disorder around the chin persisted more than 6 months after surgery in all patients. Recurrent laryngeal nerve palsy revealed in one patient. Nobody had mental nerve palsy, and no infection developed with use of preventive antibacterial tablets for 3 days.

**Conclusions:**

We developed a new method for gasless transoral endoscopic thyroidectomy with a premandible approach and anterior neck-skin lifting. TOVANS makes possible complete endoscopic radical lymphadenectomy for papillary thyroid cancer. We believe that this method is innovative and progressive and has not only a cosmetic advantage but also provides easy access to the central node compartment for dissection in endoscopic thyroid cancer surgery.

## Introduction

Endoscopic thyroidectomy is a well-established surgical technique mainly for benign thyroid nodules, follicular tumors or Graves’ disease [1]. Various surgical techniques, e.g., the direct cervical approach (anterior [2, 3] or lateral [4, 5]), extracervical access (chest wall [6, 7], breast [8], or axilla [9–11]), and a combination of approaches [12–15], have been developed in relatively recent years. In particular, an extracervical approach avoiding a cervical incision has a cosmetic advantage. Shimizu et al. reported video-assisted neck surgery (VANS) with a gasless anterior neck skin lifting method using an approach from the chest wall for endoscopic thyroidectomy [6, 7]. We began utilizing this VANS method with an anterior precordial approach in 1999 for the management of benign thyroid nodules and have had a good outcome.

Recently, natural orifice translumenal endoscopic surgery (NOTES) has been developed as a new surgical technique for abdominal surgery. With NOTES, “scarless” abdominal operations can be performed with an endoscope passed through a natural orifice (mouth, urethra, anus, etc.) and then through an internal incision in the stomach, vagina, bladder, or colon, thus avoiding external incisions or scars. If we could perform endoscopic surgery for women with thyroid disease with passage through an orifice and avoid external scarring, they would receive immeasurable cosmetic and emotional benefits.

We developed a new transoral technique of completely scarless endoscopic thyroidectomy that incorporated the concept of NOTES in a VANS-technique with gasless anterior neck skin lifting by mechanical retraction, and beginning in September 2009 have performed thyroidectomy with this technique. Recently, Witzel et al. [16] reported transoral access for endoscopic thyroid resection in a porcine model, and Benhidjeb et al. [17] reported experimental results of natural orifice surgery on the thyroid gland for human cadavers with CO_2_ insufflation. In 2009, Wilhelm et al. [18] reported the first clinical application of transoral thyroidectomy by the sublingual approach. In that same year, we developed a new transoral technique with mechanical retraction without CO_2_ insufflation by the premandible approach. This is a report of the clinical application of gasless transoral video-assisted neck surgery (TOVANS) for human thyroid disease.

## Methods of gasless transoral-video assisted neck surgery (TOVANS)

### Indications

At present, we are using TOVANS for patients with follicular tumor, symptomatic large nodular goiter, Graves’ disease, and papillary microcarcinoma without evident lymph node metastases. After patients are given a detailed explanation of the new transoral gasless endoscopic technique, they are requested to choose between the conventional chest wall approach and the new transoral approach. Written informed consent is obtained from each patient. TOVANS was approved by the institution’s review board.

### Body position

**Fig. 1:**
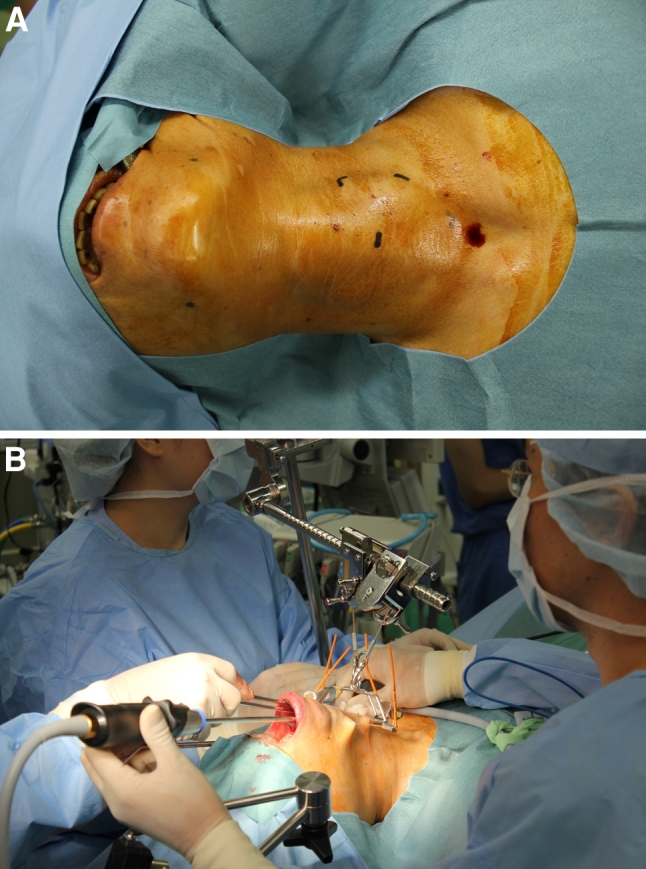
**A** Patients are placed in the supine position with neck extension. **B** *L*-shaped pole to lift up retracting wires is fixed above the patient’s neck

Under general endotracheal anesthesia, patients are placed in the supine position with neck extension created by a pillow placed under the shoulders (Fig. 1A). Because surgeons must be positioned over the head of the patient and approach the thyroid from the vestibulum under the center of the inferior lip, the endotracheal tube is fixed at the left or right corner of the mouth and equipment for anesthesia is set up on the same side of the patient. An L-shaped pole to lift up the retracting wires is fixed above the patient’s neck (Fig. 1B).

### Transoral approach and surgical procedure

**Fig. 2:**
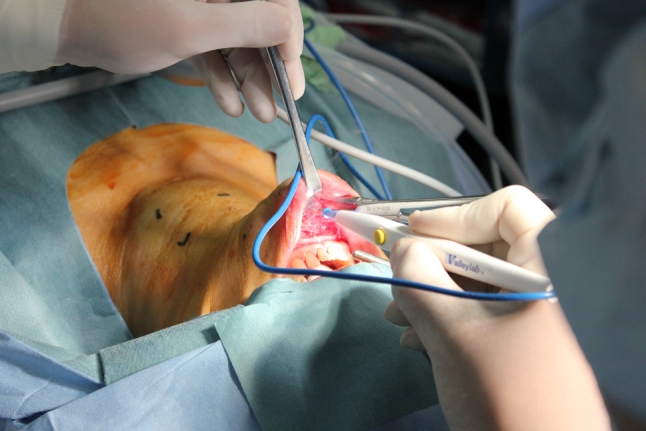
We make a 2.5 cm incision at the vestibule between the inferior lip and gingiva

Before making an incision, 40 ml of epinephrine diluted with saline is injected at the subplatysmal layer of the anterior neck and around the vestibulum oris. As a first step, we make a 2.5 cm incision at the vestibulum under the center of the inferior lip where there is a slit-like space, bounded externally by the lips and internally by the gums and teeth (Fig. 2). From the vestibulum oris to the anterior cervical region, a subcutaneous tunnel in front of the mandible is created with cotton swab forceps and electric scalpel. We keep usage of the electric scalpel and laparoscopic coagulating shears for dissection to a minimum to prevent heat damage to the mental nerve.

**Fig. 3:**
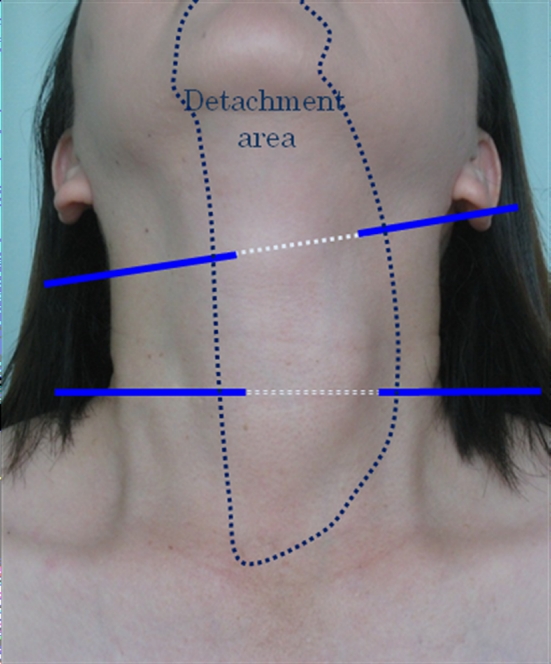
*Dotted line* shows detachment area and *blue lines* are Kirschner wires. Neck skin is lifted up mechanically by two Kirschner wire (Color figure online)

After arrival at the subplatysmal layer of the anterior neck skin, the platysma is separated from the strap muscles approximately at the level of the larynx, extending up to the suprasternal notch. Laterally, this dissection can be continued up to the medial border of the sternocleidomastoid muscle on the tumor side and up to the opposite-side border of the trachea in the case of lobectomy. In the case of total or near-total thyroidectomy, lateral dissection is performed up to the medial borderline of the sternocleidomastoid muscles on both sides. After dissection of the subplatysmal plane, two Kirschner wires with a diameter of 1.2 mm are inserted horizontally in the subplatysmal layer of the anterior cervical area (Figs. 1B, 3). Kirschner wires are lifted up by the retracting wire system fixed to the L-shaped pole above the patient’s neck (Fig. 1B). This anterior neck-skin lifting method without CO_2_ insufflation creates an effective working space and provides an excellent view, avoiding postoperative subcutaneous emphysema.

**Fig. 4:**
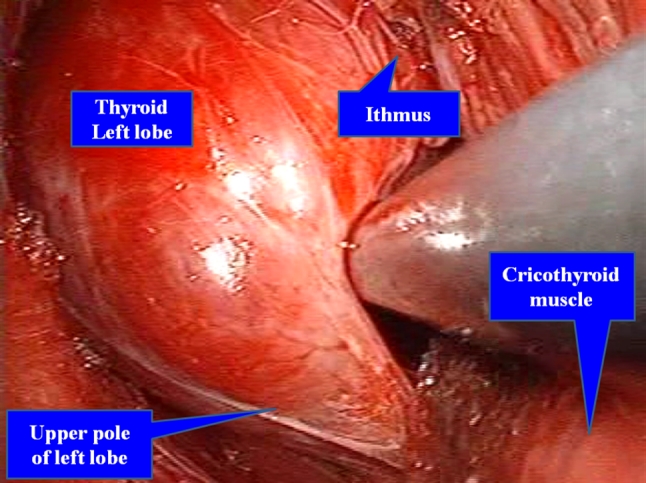
Craniocaudal view with transoral approach. After dissection of the upper pole, the superior thyroid artery and vein are divided by the Harmonic scalpel

**Fig. 5:**
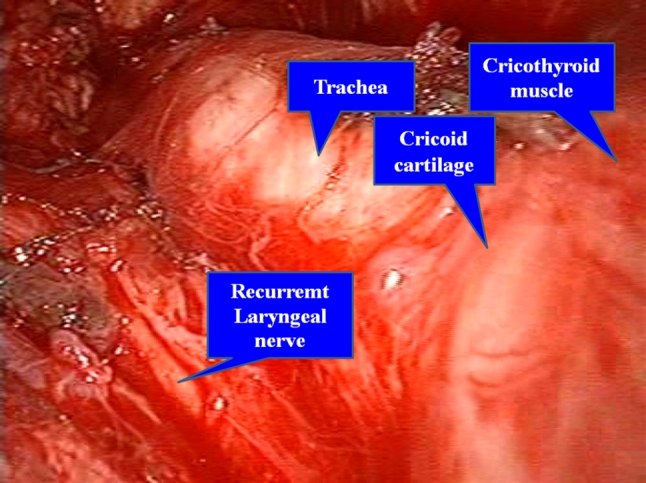
Craniocaudal view after resection of the left lobe. The recurrent laryngeal nerve is confirmed. Berry ligament is carefully divided while avoiding damage to the recurrent nerve

**Fig. 6:**
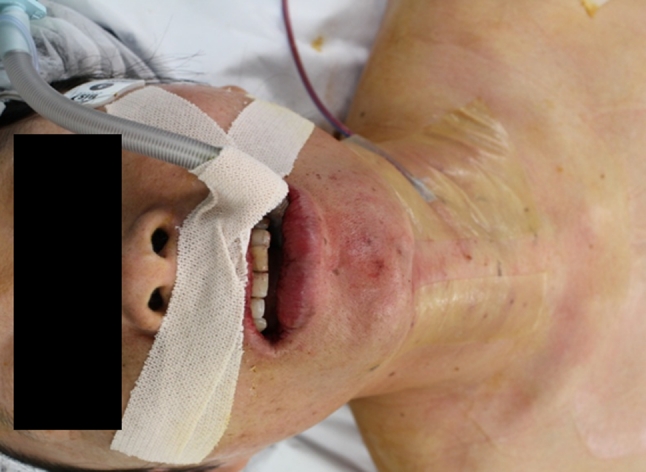
Slender drainage tube is placed beside the trachea. Drainage tube may not always be necessary

Avoiding the sternothyroid muscles laterally, the thyroid gland is revealed. The isthmus is transected with a Harmonic scalpel after blunt dissection of the thyroid gland from the trachea. Following the dissection of the upper pole, the superior thyroid artery and vein are divided (Fig. 4). The medial thyroid vein is also cut off by the Harmonic scalpel. After preparation of the retrothyroidal area, the recurrent laryngeal nerve is confirmed with certainty and the inferior thyroid artery is divided. The Berry ligament is then carefully divided while avoiding damage to the recurrent nerve, and thereafter the thyroid gland is removed (Fig. 5). In a total or near-total thyroidectomy, we perform this procedure in both sides. To be on the safe side, a slender drainage tube is placed beside the trachea (Fig. 6). Incisions in the mouth are closed with absorbable sutures.

## Results

**Fig. 7:**
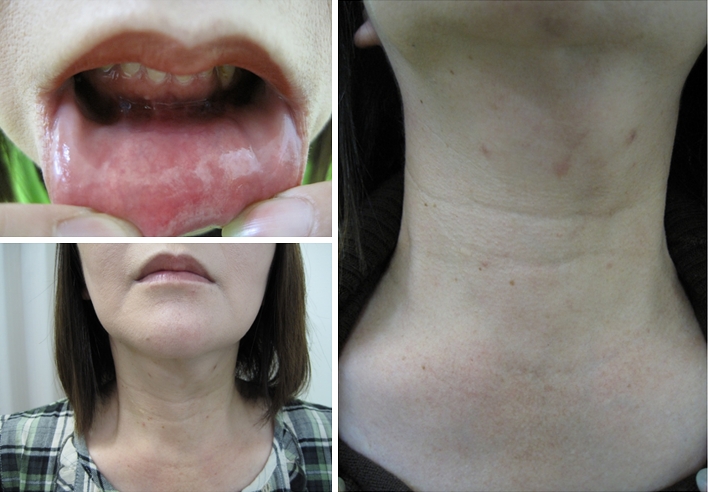
Photograph one month after surgery. *Incisional wound* in the mouth has healed. There is no surgical scar on the patient’s neck without a drain hole

Beginning with our first clinical application of TOVANS in September 2009, we have performed eight such procedures. Three of the eight patients had papillary microcarcinoma and received central node dissection after thyroidectomy (Table 1). Operative time of simple hemithyroidectomy in five patients ranged from 151 to 233 (average 208) min, and of subtotal thyroidectomy with central node dissection in three patients ranged from 310 to 414 (average 361) min. Despite the small number of patients, operative time of latest case had become quite a bit shorter than that of first case. The average amount of blood and fluid lost was around 97 ml with an amount between 5 and 225 ml in this surgical procedure. All patients began oral intake 1 day after surgery, and the slender drain was removed the same day. They left the hospital on the fourth or fifth day after the operation. Although the lower lip was swollen for several days after the operation, it healed naturally. A photograph taken 1 month after surgery is shown in Fig. 7. By this time, patients had recovered completely and had the same appearance as before the operation. The sensory disorder around the chin persisted for more than 6 months after surgery in all patients. Recurrent laryngeal nerve palsy was revealed in one patient. Nobody had mental nerve palsy, and no infection developed with use of preventive antibacterial tablets for 3 days.

**Table 1:** Surgical features of patients with TOVANS

Patient	Diag.	Resection	Dissection	Ope. time	Bleeding	Per Os	Hospital stay	Complication
50F	FA	Lob	–	3:53	130	1	4	–
55F	FA	Lob	–	3:37	<10	1	4	–
55F	NG	Lob	–	3:48	225	1	5	–
*68M*	*PC*	*Sub*	*CND*	*6:54*	*100*	*1*	*5*	*RLN palsy*
*57F*	*PC*	*Sub*	*CND*	*6:00*	*110*	*1*	*4*	*–*
31F	FA	Lob	–	3:34	130	1	4	–
*68F*	*PC*	*Sub*	*CND*	*5:10*	*30*	*1*	*4*	*–*
55F	FC	Lob	–	2:31	45	1	4	–

*FA* follicular adenoma, *NG* nodular goiter, *PC* papillary carcinoma, *Lob* lobectomy, *Sub* subtotal thyroidectomy, *CND* central node dissection, *RLN* recurrent laryngeal nerve, *Italics* indicate the patient with papillary thyroid carcinoma

## Discussion

Endoscopic thyroid surgery for thyroid nodules or Graves’ disease has been developed in this decade. Replacing the area of incision from the anterior cervical area to the precordial or axillary region has provided cosmetic benefits to patients, especially young women. We have used endoscopic thyroidectomy with the gasless precordial approach since 1999 for the management of benign thyroid nodules and have had a good outcome. The concept of NOTES, which was recently introduced to laparoscopic surgery, was novel from the viewpoint of minimal incision surgery.

Witzel et al. [16] and Benhidjeb et al. [17] reported a simulation of transoral thyroidectomy with CO_2_ insufflation approached from the sublingual floor of the mouth. We originally developed a procedure for gasless transoral endoscopic thyroidectomy using the mechanical retraction method approaching the thyroid from the premandible route with an incision at the vestibular oris. Our transoral method has two major features. One is that a working space is created by mechanical lifting without CO_2_ insufflation, which provides an excellent endoscopic view during the operation, avoiding postoperative emphysema. The other is that only small route is created to the thyroid gland from a subcutaneous tunnel of the vestibular oris, which provides a safe procedure with little risk of mental nerve injury. Through our experience with this new gasless transoral method for thyroidectomy, we were able to demonstrate its safety and feasibility as ultimate minimal incision surgery. In Japan, surgery-related hospitalization costs burden on the patients is not so expensive. Therefore, many Japanese patients want to be in hospital as long as possible. In this series, all of the patients left the hospital on the fourth or fifth day after the operation. In fact, they all began oral intake 1 day after surgery and the slender drain was removed the same day. So, it is probable that all patients could be discharged from the hospital 1 day after surgery.

When performing this procedure, it is important to give special attention to preventing thermal damage to the mental nerve. It is desirable to confirm preoperatively the position of the mental foramen with multidetector-row computed tomography. During dissection of the subcutaneous area around the chin, to avoid heat damage, we minimized the use of the electric knife or Harmonic scalpel. Because of small number of patients, TOVANS still takes a long surgical time. From our experience thus far, it can be considered that the operative time will become still shorter as cases are accumulated.

In addition to its usefulness for thyroidectomy, this transoral approach is valuable for complete dissection of pretracheal and laterotracheal lymph nodes. With the transaxillary approach or precordial approach, dissection of the paratracheal lymph nodes behind the sternum is likely to be inadequate. However, because we can get an excellent craniocaudal view by the transoral approach, access to pretracheal and laterotracheal lymph nodes is easy. We have already performed complete central node dissection in three patients with papillary thyroid microcarcinoma. We consider that the operation with craniocaudal view is the most important factor for endoscopic complete central node dissection; therefore, TOVANS is a very useful surgical procedure for complete endoscopic central node dissection. Furthermore, complete endoscopic radical surgery for malignant thyroid tumors not only with central node lymphadenectomy but also with lateral node dissection may be possible in the near future.

## Conclusions

We have described a new method for gasless transoral endoscopic thyroidectomy with a premandible approach and anterior neck-skin lifting. TOVANS makes possible complete endoscopic radical lymphadenectomy for papillary thyroid cancer. We believe that this method is innovative and progressive and has not only a cosmetic advantage but also provides easy access to the central node compartment for dissection in endoscopic thyroid cancer surgery.
